# Deep-learning-based extraction of circle of Willis topology with anatomical priors

**DOI:** 10.1038/s41598-024-80574-0

**Published:** 2024-12-30

**Authors:** Dieuwertje Alblas, Iris N. Vos, Micha M. Lipplaa, Christoph Brune, Irene C. van der Schaaf, Mireille R. E. Velthuis, Birgitta K. Velthuis, Hugo J. Kuijf, Ynte M. Ruigrok, Jelmer M. Wolterink

**Affiliations:** 1 Department of Applied Mathematics, Technical Medical Centre, University of Twente, Enschede, The Netherlands; 2 Image Sciences Institute, University Medical Center Utrecht, Utrecht, The Netherlands; 3 Department of Biomedical Engineering, Eindhoven University of Technology, Eindhoven, The Netherlands; 4 Department of Radiology, University Medical Center Utrecht, Utrecht, The Netherlands; 5 Department of Neurology and Neurosurgery, University Medical Center Utrecht, Utrecht, The Netherlands

**Keywords:** Circle of Willis, Vessel tracking, Anatomical priors, Deep Learning, Neuro-vascular interactions, Computer science, Biomedical engineering

## Abstract

The circle of Willis (CoW) is a circular arrangement of arteries in the human brain, exhibiting significant anatomical variability. The CoW is extensively studied in relation to neurovascular pathologies, with certain anatomical variants previously linked to ischemic stroke and intracranial aneurysms. In an individual CoW, arteries might be absent (aplasia) or underdeveloped (hypoplasia, diameter < 1 mm). As the assessment of such variations is time-consuming and susceptible to subjectivity, robust automatic extraction of personalized CoW topology from time-of-flight magnetic resonance angiography (TOF-MRA) images would highly benefit large-scale clinical investigations. Previous work has sought to extract CoW topology from voxel-based semantic segmentation masks. However, hypoplastic arteries are challenging to recover in voxel-based segmentation. Instead, we propose using a complete CoW as an anatomical prior for extracting all possible CoW arteries as shortest paths between automatically identified anatomical landmarks, guided by automatically determined artery orientation vector fields. These fields are obtained using a scale-invariant and rotation-equivariant mesh-CNN-based model (SIRE). For a 3D TOF-MRA volume, a potentially overcomplete graph of the CoW is thus extracted in which each edge represents an artery. Subsequently, a binary Random Forest classifier labels each artery as normal or hypo-/aplastic. The model was optimized and validated using a data set of 351 3D TOF-MRA scans in a cross-validation setup. We showed that using a shortest path algorithm with a cost function based on local artery orientations results in continuous artery paths, even in hypoplastic cases. We tracked the correct path in the posterior communicating arteries in 70–74% of the cases, an artery that is known to pose challenges in voxel-based segmentation models. Our downstream artery path classifier obtained an average F1 score of 0.91, demonstrating the potential of our proposed framework to extract personalized CoW topology automatically.

## Introduction

**Figure 1:**
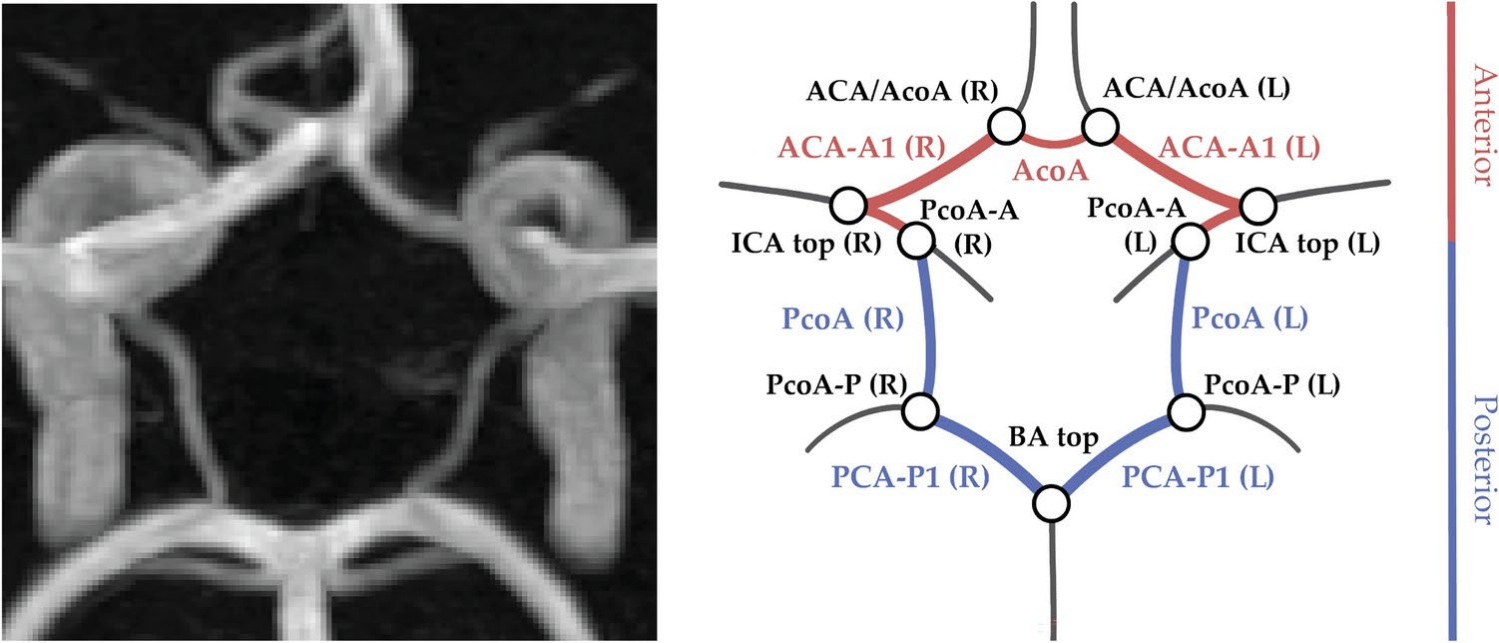
Maximum intensity projection of a 3D TOF-MRA scan (left) and schematic representation (right) of a complete and symmetrical CoW. Arteries in the anterior and posterior section of the CoW are shown in pink and blue respectively, bifurcations are shown in black. All artery and bifurcation labels are included.

The circle of Willis (CoW) is a circular arrangement of arteries located at the base of the brain, responsible for regulating cerebral blood flow. Variations in the configuration of the CoW are prevalent: it is estimated that only less than a quarter of the general population has a complete and symmetrical CoW^1^, see Fig. 1. Some anatomical variations have been associated with neurovascular diseases, such as ischemic stroke and intracranial aneurysms^2–4^. However, these findings still lack sufficient evidence for clinical implications, and methodological approaches to assess CoW variation can substantially differ between studies^4,5^. Common variations include hypoplasia (underdevelopment) or aplasia (absence) of one or both posterior communicating arteries (PcoAs). Definitions of hypoplasia vary from artery diameters below 0.5 to below 1.0 mm in literature, contributing to a wide range of reported CoW variation prevalences^5^. In addition, the use of classification systems to categorize CoW variants is inconsistent: studies may rely on established systems, e.g. Lippert & Bapst^6^ or Lazorthes^7^, deviations from these systems, or no system at all. These discrepancies are further amplified by the intra- and inter-subject variability introduced by manual diameter measurements taken at a single point to identify hypoplasia.

The CoW vasculature can be imaged non-invasively through 3D time-of-flight magnetic resonance angiography (3D TOF-MRA). In this modality, local blood flow produces high image intensities, resulting in a clear contrast between arteries and surrounding tissues in the scan (Fig. 1, left). To achieve a standardized, reproducible assessment of the CoW, automatic methods are required that are capable of recovering its topology from the 3D TOF-MRA’s, despite its large and inherent variability. These methods could facilitate large-scale analyses across populations, enabling further investigation into the relationship between anatomical variations and neurovascular diseases. Previous attempts to recover the topology of the CoW automatically have commonly relied on artery segmentations obtained through classical image processing methods^8,9^ or deep learning algorithms^10,11^. These artery segmentations are typically represented as binary classifications for each voxel in the 3D scan, i.e. a voxel-mask. However, the desired continuous, tubular topology is challenging to impose on these voxel-mask segmentations, as they may contain holes or disconnected parts. This problem is especially evident for smaller arteries of the CoW. A review of the results obtained in the TopCoW’23 challenge, which focused on anatomical segmentation of the CoW, found that segmentation of artery segments with diameters around 1 mm is an open problem^12^. This raises the broader question of whether segmentation-based methods can reliably capture the CoW topology. Hypoplastic arteries often have diameters smaller than the voxel size in typical clinical settings and can be invisible in some slices of the 3D scan. Any algorithm depending on identification of individual voxels, is thus unlikely to reliably reconstruct these hypoplastic arteries. Alternatively, artery centerlines and graph representations can be used as a starting point for automatic analysis of cerebrovascular networks containing the CoW. This approach has been successfully used to perform labeling in the CoW^13^, connectivity aware segmentation^14^ and topology extraction of cerebrovascular networks^15^.

In this work, we use artery trajectories as a starting point^13–15^ to automatically obtain the CoW topology as a graph representation. We overcome the limitations of voxel-based segmentation for the CoW by proposing a method that provides a continuous (over)complete representation of the CoW. We impose the structure of the CoW by using path optimization between automatically identified bifurcations. Path optimization algorithms have been used to extract arterial segments in the CoW but typically rely on a (sparse) graph representation from the skeletons derived from segmentation masks^8,16^. However, as a consequence of discontinuous skeletons, hypoplastic arteries may not be connected in this graph representation. Here, we propose to perform path optimization on the Cartesian image grid, using a learned cost function based on local artery orientations to find correct and continuous artery trajectories. By classifying each resulting trajectory according to Lippert’s system as ’normal’ or ’hypo-/aplastic’, we expect our method improves the inclusion of hypoplastic arteries, enhancing the accuracy and reliability of large-scale CoW anatomical assessments.

## Materials and methods

**Figure 2:**
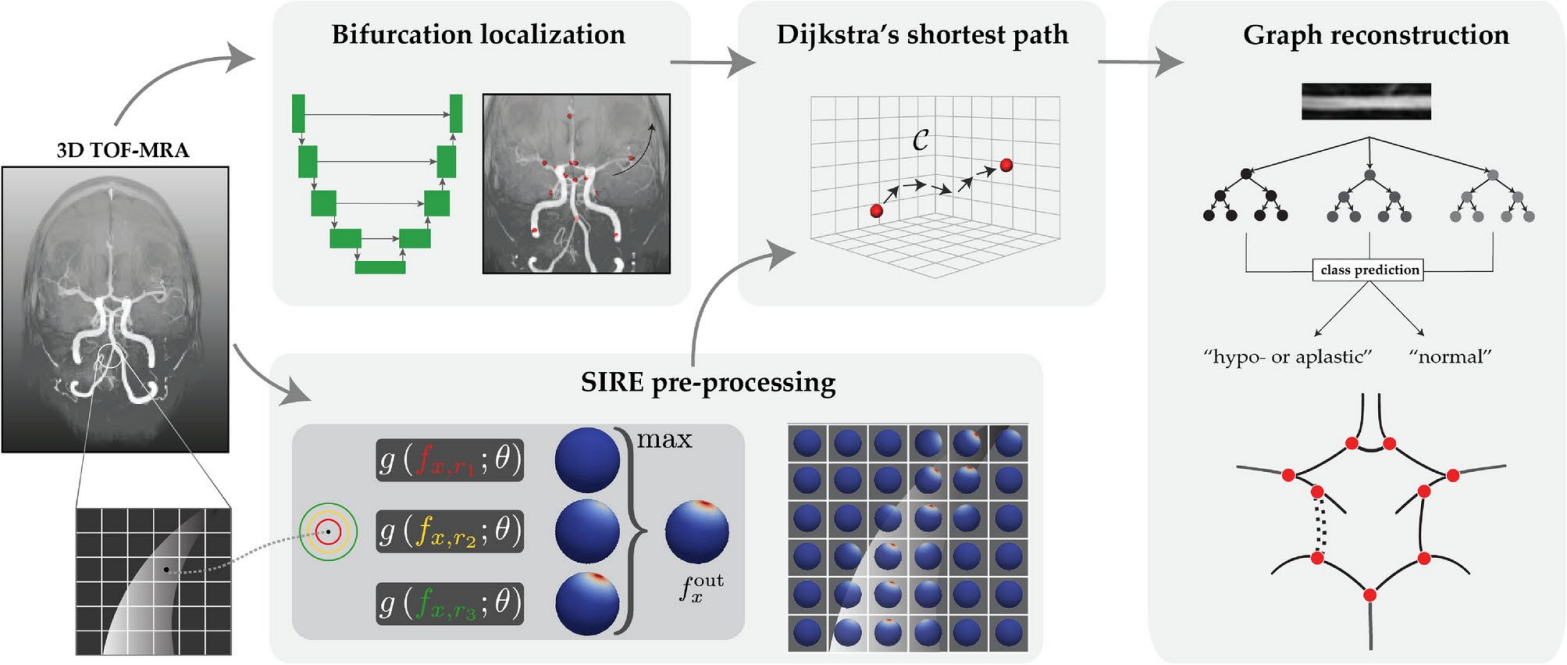
Method overview. CoW bifurcation points were detected on a TOF-MRA scan. Local artery orientations were estimated and embedded into a cost function $\mathcal {C}$. For each connection between bifurcation points in a complete CoW, the shortest path was found based on $\mathcal {C}$. Paths were classified as *normal* or *hypo-/aplastic* using intensity profiles and used for a graph representation of the CoW.

We propose to extract the CoW topology as a graph representation by automatically acquiring an (over)complete CoW from a 3D TOF-MRA scan. We consider the topology to consist of seven arterial segments that connect at nine bifurcations. These segments are (Fig. 1): the anterior communicating artery (AcoA), and the left and right A1-segments of anterior cerebral (ACA-A1), P1-segments of posterior cerebral (PCA-P1), and posterior communicating (PcoA) arteries. Figure 2 gives an overview of our method, consisting of four consecutive steps. First, the the nine CoW bifurcation points were automatically detected. Second, we obtained local artery orientations at each point in a grid containing the CoW. Third, CoW bifurcation points were automatically connected by a shortest path algorithm using a cost function based on these local artery orientations. Fourth, each reconstructed path was classified as ‘normal’ or ‘hypo-/aplastic’. This resulted in a graph representation of the CoW topology, following Lippert’s classification system^6^. Our code is publicly available (https://github.com/MIAGroupUT/CoW_topology_extraction).

### Data

We included 3D TOF-MRA scans of 351 healthy individuals who were part of a research screening cohort conducted by the University Medical Center Utrecht, the Netherlands. All individuals received a 3D T1-weighted gradient echo TOF-MRA acquisition on either a 1.5T or 3T MR-scanner (Philips Healthcare, Best, The Netherlands). The in-plane resolution of these scans ranged between 0.20 × 0.20 mm^2^ and 0.39 × 0.39 mm^2^, with a slice thickness of 0.5 mm. The dimensions of the 3D TOF-MRA scans ranged between 512 × 512 × 140 and 576 × 576 × 172 voxels.

We acquired manual annotations of nine bifurcation positions and seven artery diameters as shown in Fig. 1 (*right*) in all scans. Artery diameters were measured at a single position. We manually annotated centerlines for the normally developed and hypoplastic artery segments in 31 selected scans. Centerlines of aplastic artery segments were omitted. Two experienced (neuro)radiologists annotated artery diameters, while two observers, trained by one radiologist, annotated bifurcation positions and centerlines. Arteries were categorized as ’normal’ ($\ge$ 1.0 mm) or ’hypo-/aplastic’ (< 1.0 mm) based on the manually measured diameters.

### Artery bifurcation localization

We cast the automatic detection of arterial bifurcation points as a regression problem, where each annotated bifurcation point was transformed to a discretized scalar field (heatmap), whose local maximum corresponds to the bifurcation location^17^. Ground-truth heatmaps were generated by convolving each bifurcation point with a Gaussian kernel ($\sigma$ = 5 voxels). We trained a 3D U-Net to predict the heatmap for each of the nine bifurcation points based on the 3D TOF-MRA scan^18^. As pre-processing steps, we resampled all scans to a uniform voxel size of ($0.36 \times 0.36 \times 0.50$) mm, cropped the scans to a size of $256\times 256\times 128$ around the center, normalized the image intensities using z-score normalization and corrected shadowing artefacts using N4 bias field correction. The network was trained using a mean absolute error loss, using an Adam optimizer with a learning rate of $1\text {e}^{-4}$ and a batch size of 1 on an NVIDIA Titan X (12 GB) GPU for 85 epochs. No data augmentation was used.

### Local artery orientation estimation

We connect the automatically identified bifurcation points using a cost function that incorporates local artery orientations. This approach is expected to produce more accurate artery trajectories, even in cases of underdeveloped arteries (hypoplasia). We defined the cost function on a 3D Cartesian grid $\mathcal {G}$ with a 0.5 mm spacing that contains the CoW. At each point on this grid, we estimated the local artery orientations from local image information using a graph neural network $g(\cdot ; \theta )$.

Artery orientation estimation was treated as a regression problem on the surface of a discretized sphere. The *N* local artery directions $\{\varvec{d}_i\}_{i=1,...,N}$ at $x\in \mathbb {R}^3$ were represented as the locations of the top-*N* maxima on the scalar field $f^{\text {out}}_x$ on the surface of a sphere. As we assumed no branching of artery trajectories between the detected bifurcation points, we took the top-2 local maxima to find artery orientations $\varvec{d}_1$ and $\varvec{d}_2$ at each $x \in \mathcal {G}$.

Given the varying diameters and orientations of CoW arteries, the artery orientation estimator must be robust to these differences. The estimator we used, SIRE^19^, achieved this by being both scale-invariant and rotation-equivariant. This means that the estimated orientations remain accurate despite changes in artery diameter and direction.

SIRE is insensitive to variation in artery diameter, as it processes nested image patches of multiple sizes centered at *x* in *parallel* ({$r_1$, $r_2$, $r_3$} in Fig. 2). $g(\cdot ; \theta )$ predicted the scalar field $f^{out}_{x,r_i}$ for the image patch of each size. These scale-wise outputs were aggregated through a vertex-wise maximum operation, resulting in the final output $f^{\text {out}}_x$ from which $\varvec{d}_1$ and $\varvec{d}_2$ were inferred. As a result of this multi-scale approach, the estimator based its orientation estimation on image patches of adequate size with respect to the diameter of the artery while ignoring information from other image patches.

To achieve rotational equivariance, the orientation estimation was performed intrinsically on a sphere. Local image data around each *x* within a distance *r* was projected onto the sphere’s surface, resulting in the spherical input patch $f_{x,r}$. These image patches were processed by a graph neural network $g(\cdot ; \theta )$, which operates on the surface of a sphere and predicts scalar output $f^{\text {out}}_{x,r}$. This approach results in rotational equivariance; rotation of the artery orientation in the image leads to a similar rotation of the image projection on the spherical surface, resulting in a rotated prediction $f^{\text {out}}_{x,r}$.

The network $g(\cdot ;\theta )$ was trained using manually annotated centerlines. Points *x* were randomly sampled on these centerlines and multi-scale image patches $f_{x,r}$ were constructed based on the patch sizes manually defined before training. Additionally, a ground-truth response on the spherical surface was constructed from the manual centerline. The weights $\theta$ of $g(\cdot ;\theta )$ were updated using the mean squared error loss between $f^{\text {out}}_x$ and this ground-truth response. We used an Adam optimizer with a learning rate of 0.001 and a batch size of 10 for 5,000 epochs to train $g(\cdot ; \theta )$ using image patch sizes of $\{1, 2, 5, 7, 10 \}$ mm.

The orientation estimation procedure above assumes that *x* is located *inside* the artery lumen. However, for many points $x \in \mathcal {G}$ this is not the case. When *x* is not inside the artery lumen, the activation values on $f^{\text {out}}_x$ are expected to be more spread out and have less pronounced maxima. This can be quantitatively measured as the entropy of $H\left( \cdot \right) : f^{\text {out}}_x \rightarrow [0, 1]$, which is calculated as:

1 $$\begin{aligned} H\left( f^{\text {out}}_x \right) = - \frac{1}{\ln (|\mathcal {V}|)} \sum \limits _{v\in \mathcal {V}} f^{\text {out}}_x(v) \ln \left( f^{\text {out}}_x(v) \right) . \end{aligned}$$

An entropy value close to 1 indicates a more dispersed scalar field $f^{\text {out}}_x$, reflecting less certainty in the artery orientation at that point, indicating that *x* was not inside the artery lumen. During training, this behaviour is encouraged by sampling points *x* outside the artery lumen with a probability $p=0.3$ and a zero ground truth response, which yields an entropy of 1. We used segmentation masks for this sampling strategy.

### Path extraction

We used the outputs $f^{\text {out}}_x$ at every point $x \in \mathcal {G}$ to construct cost functions to connect the previously found bifurcation points in the CoW using a path optimization algorithm. We ensured efficient calculations of the local artery orientations by batching. This enables creation of nested, spherical input patches and the estimation of the artery orientations for 500 points on the Cartesian grid $\mathcal {G}$ in parallel. To extract accurate CoW trajectories, cost functions should be low inside the artery lumen and high outside. From $f^{\text {out}}_x$, we computed the two artery orientations $\varvec{d}_1$ and $\varvec{d}_2$ at every point, as well as the entropy $H\left( f^{\text {out}}_x \right)$. We constructed two different cost functions from the artery orientations and entropy of $f^{\text {out}}_x$, that are denoted as $\mathcal {C}_{\text {orient}}$ and $\mathcal {C}_{\text {entr}}$, respectively:

2 $$\begin{aligned} \mathcal {C}_{\text {orient}}(x)&= \frac{1}{2} \left( 1 - \text {mean cossim } \mathcal {N}(x)\right) , \end{aligned}$$

3 $$\begin{aligned} \nonumber \text {mean cossim } \mathcal {N}(x)&= \frac{1}{26} \sum \limits _{y \in \mathcal {N}(x)} \frac{1}{2} \max \left( \langle \varvec{d}_1^x, \varvec{d}_1^y \rangle + \langle \varvec{d}_2^x, \varvec{d}_2^y \rangle , \langle \varvec{d}_1^x, \varvec{d}_2^y \rangle + \langle \varvec{d}_2^x, \varvec{d}_1^y \rangle \right) \\ \mathcal {C}_{\text {entr}}(x)&= H\left( f^{\text {out}}_x\right) . \end{aligned}$$

The cost function $\mathcal {C}_{\text {orient}}$ was based on local artery orientations, representing the consensus in the predicted orientations at *x* ($\varvec{d}_i^x$) and at its 26 neighbours $y \in \mathcal {N}(x)\subset \mathcal {G}$ in terms of the cosine similarities ($\langle \cdot , \cdot \rangle$). As the two predicted artery orientations are unordered, we take the maximum cosine similarity of both possible combinations. Note that there was no ordering of the two predicted directions at each point hence we took the maximum. We expect similar predicted orientations for *x* and its neighbours *y* inside the artery lumen, resulting in cosine similarities close to one and thus a low cost function. As the entropy of $f^{\text {out}}$ is low inside the artery lumen and high outside, $\mathcal {C}_\text {entr}$ also exhibits low values inside and high values outside the artery lumen.

Additionally, to demonstrate the merit of an orientation-guided cost function as we propose in this work, we constructed a cost function based on local image intensities, which we use as a baseline. We first normalized the image intensities in each 3D TOF-MRA to the [0,1] domain between the 1^st^ and 99^th^ intensity percentiles of each individual scan. We defined the image-guided cost function denoted by $\mathcal {C}_{\text {img}}$ as:

4 $$\begin{aligned} \mathcal {C}_{\text {img}}(x) = - I(x), \end{aligned}$$

where *I*(*x*) is the image intensity at *x*, obtained by trilinear interpolation. Since image intensities are higher inside the artery lumen than in surrounding tissues, our cost function $\mathcal {C}_{\text {img}}$ has low values inside the lumen and high values outside, as desired. We created a fourth cost function $\sum \mathcal {C}_i$, that is the sum of the cost functions $\mathcal {C}_{\text {orient}}$, $\mathcal {C}_{\text {entr}}$ and $\mathcal {C}_{\text {img}}$.

To extract the artery paths, we used Dijkstra’s shortest path algorithm^20^ on the Cartesian grid $\mathcal {G}$ subject to one of the four cost functions as defined above. Dijkstra’s algorithm computes the optimal path between a given seed point and sink point. Here, we considered the bifurcation points in a complete CoW configuration (Fig. 1) as seed and sink points. For each artery segment, we used the corresponding bifurcation points to find a minimal cost path.

Finally, we compared the merits of our method that finds connected artery trajectories with those obtained from a voxel-mask segmentation of the CoW vasculature. Using a 3D U-Net architecture for voxel-mask segmentations^21^, we derived trajectories through skeletonization and compared these to the trajectories obtained using the proposed method.

### Graph reconstruction

The paths resulting from our path extraction step were used to reconstruct a graph-based representation of the CoW topology. Following Lippert’s classification system^6^, we automatically classified each path as either ’normal’ or ’hypo-/aplastic’, with hypoplasia defined as artery diameters < 1mm. We trained a Random Forest classifier (RF) using intensity profiles along each path, which we resampled to a fixed size of 50 to use as input features. The RF classifier was implemented using the scikit-learn library, with the maximum tree depth set to 2 to prevent overfitting, the number of decision trees set to 100 and using balanced class weights. The classified paths (edges), along with predicted bifurcation locations (nodes), form a graph representation of the CoW.

### Evaluation criteria

To assess each step of our multi-scale approach for automatic CoW topology extraction, we employed both quantitative and qualitative criteria. We measured the performance of our automatic bifurcation localization method by calculating the Euclidean distance between manually annotated and automatically detected bifurcation points.

For the quality of the extracted artery trajectories, we used the Fréchet distance to compare our results to the manually annotated centerlines, focusing on both normal and hypoplastic arteries. As we cannot measure these distances for arteries that are absent, aplastic segments were excluded from quantitative evaluation. We qualitatively assessed if our method followed the intended artery trajectory.

We assessed the performance of our RF classifier using the F1 score. Additionally, we assessed the accuracy of the CoW topology representations by comparing the Lippert class resulting from our graph representation to the class determined by a medical expert. Lippert classifies the anterior and posterior sections of the CoW separately and differentiates between normally developed or hypo-/aplastic arterial segments. We include three Lippert classes for the anterior section and five classes for the posterior section. A schematic overview with the developed artery sections for each class is given in Fig. 5, where solid and dashed lines represent normally developed and hypo-/aplastic segments, respectively. Given our focus on hypo-/aplasia, we did not account for Lippert classes involving CoW variations such as additional arteries or fetal-type PCA^22^. Lippert classes involving these variation types are combined or excluded from our analysis.

**Figure 3:**
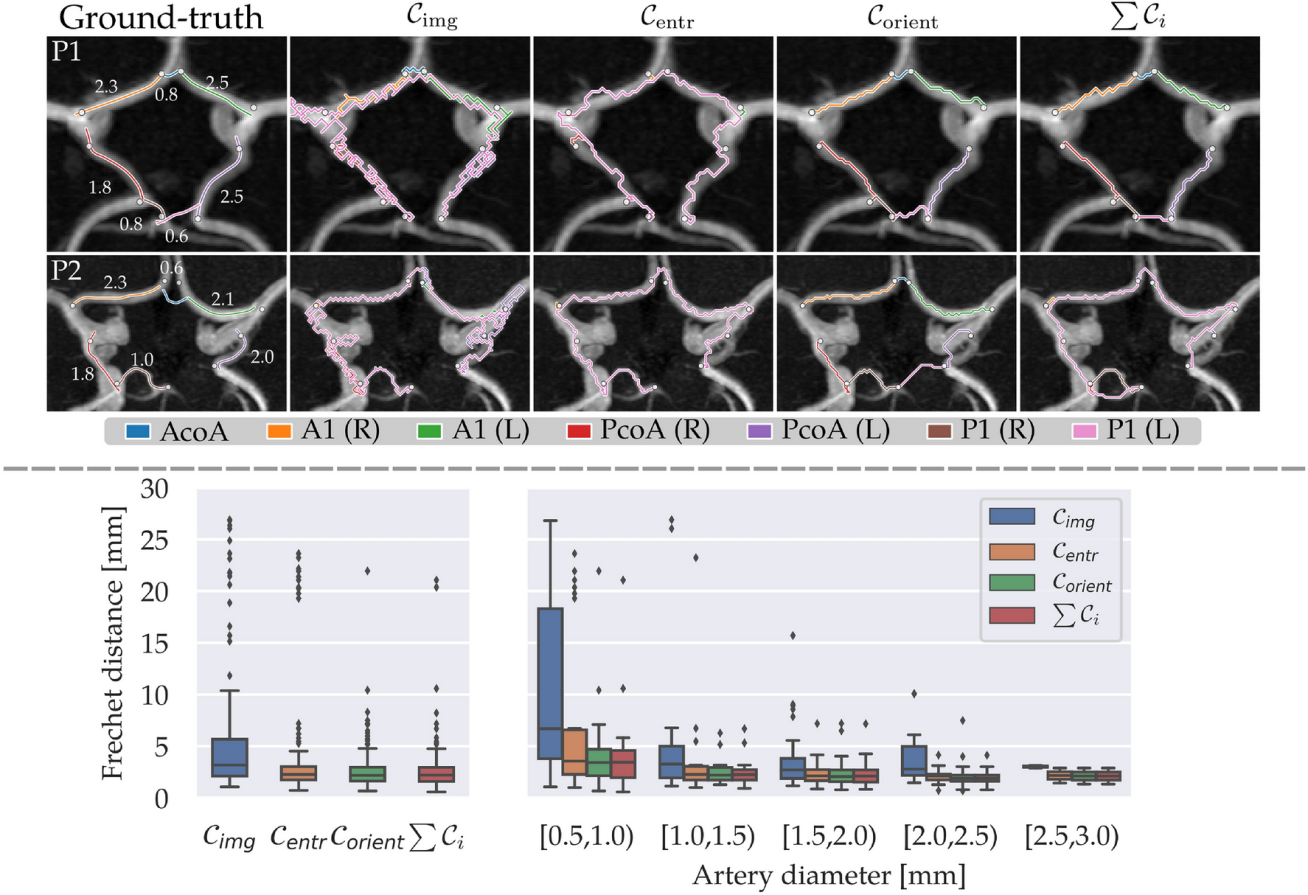
Results of our path extraction experiments. *Top:* Qualitative results showing maximum intensity projections of the 3D TOF-MRA around the CoW, with 2D projections of the centerlines and automatically detected bifurcation points for two patients. Manually annotated centerlines, with measured diameters in mm (*left*). Centerlines from Dijkstra’s algorithm using $\mathcal {C}_{\text {img}}$, $\mathcal {C}_{\text {entr}}$ and $\mathcal {C}_{\text {orient}}$ (*right*). *Bottom: * Quantitative results showing Fréchet distances between manual centerlines and lines acquired using $\mathcal {C}_{\text {img}}$, $\mathcal {C}_{\text {entr}}$, $\mathcal {C}_{\text {orient}}$ and $\sum \mathcal {C}_i$ for all arteries (*left*) and categorized by artery diameter (*right*).

## Experiments and results

Our dataset consisted of 351 3D TOF-MRA’s, that were split into 311 cases for training, and 40 for testing. To train and evaluate our network for automatic bifurcation detection, we performed 6-fold cross validation on 300 of the training cases. For training our artery orientation estimator, we used the remaining 11 training cases and used 20 cases from the test set with manually annotated centerlines to assess the quality of the extracted CoW trajectories. Our RF classifier relies on intensity features along the automatically extracted CoW trajectories resulting from the automatic bifurcation detection and the local artery orientation estimation. We trained the RF classifier on 300 scans, with 250 scans allocated for training and 50 for validation. To avoid data leakage, we trained the RF classifier using the intensity features from the extracted paths from the validation set of each of the six cross validation folds. The data splits are visualized in Figure S1 in Appendix A (Supplementary).

### Artery bifurcation localization

We combined the six trained models resulting from cross-validation into an ensemble and evaluated the localization performance of this ensemble model on our held-out test set. For all nine bifurcations, the median distance between the automatically detected and the annotated bifurcation point was below 1.9 mm. We observed outliers up to 7.1 mm. Lowest performances were obtained for the PcoA-P (left and right), with median distances of 1.6 mm and 1.8 mm, respectively. The best performance was observed for the BA top bifurcation point, that had a median distance of 0.76 mm. We expect that this performance discrepancy is related to the variation in CoW configurations. For example, for an aplastic PcoA(R) segment, the location of the PcoA-P(R) bifurcation is ambiguous. In contrast, the BA top bifurcation is easier to distinguish in the case of hypo-/aplastic segments. Figure S2 (Appendix B, Supplementary) shows a boxplot of the detection performance for each of the nine bifurcations.

**Figure 4:**
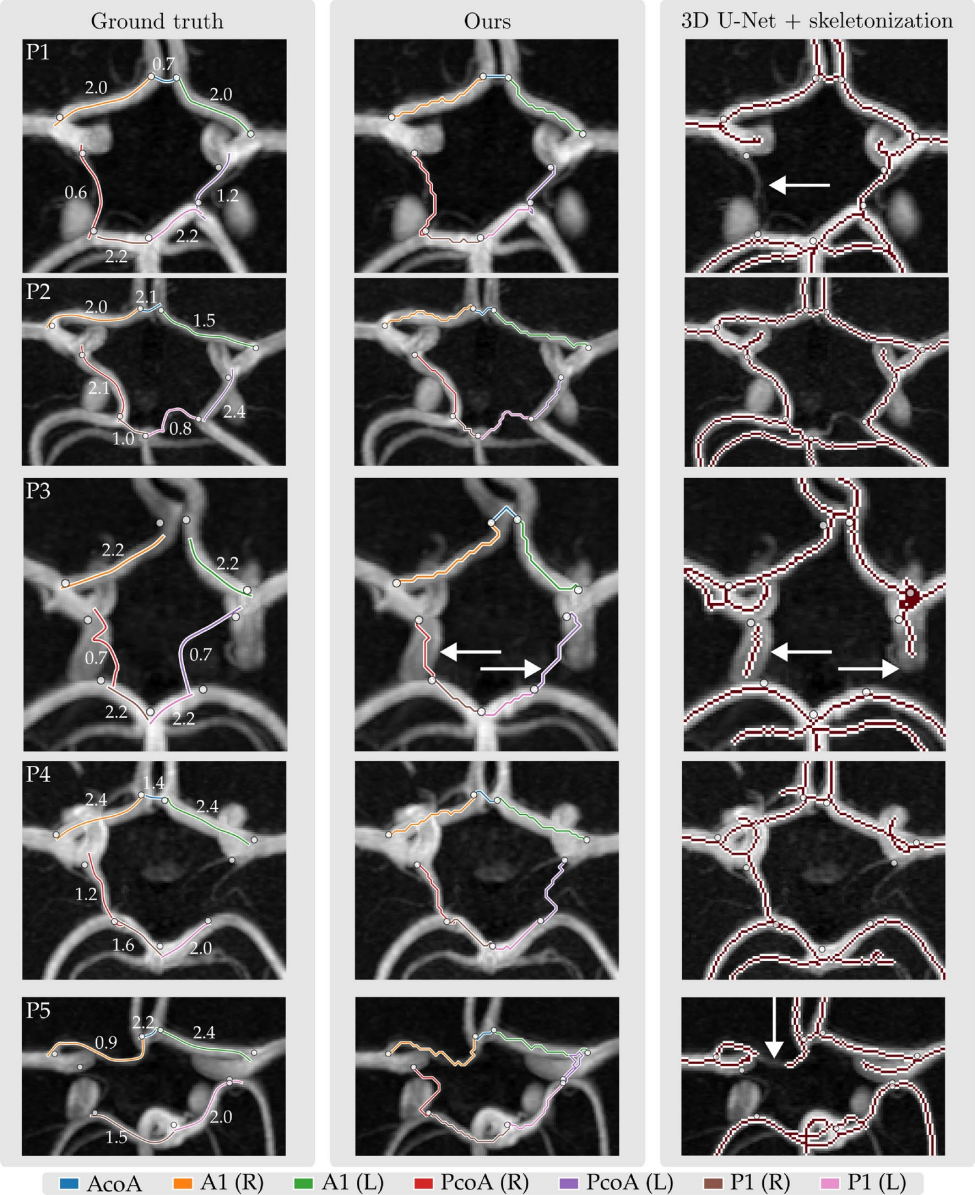
Comparison between path localization using our proposed framework using $\mathcal {C}_{\text {orient}}$(middle column) and skeletonization on a 3D U-Net segmentation (right column). The 3D U-Net does not find continuous trajectories for hypoplastic arteries, while our method finds a continuous path by design. Our method sometimes traverses the wrong artery segment. Both errors are indicated with a white arrow.

### Path extraction

We used Dijkstra’s algorithm to connect the detected artery bifurcations using the four cost functions $\mathcal {C}_{\text {img}}$, $\mathcal {C}_{\text {entr}}$, $\mathcal {C}_{\text {orient}}$ and $\sum \mathcal {C}_i$. Figure 3 (top) shows the extracted CoW artery trajectories using the four cost functions, as well as the manually annotated centerlines and measured diameters. We observed that Dijkstra’s algorithm struggles to find a plausible connection for hypo-/aplastic arteries when cost functions that do not include artery orientation. In these cases, paths traverse the CoW in the opposite direction to connect bifurcation points. Moreover, paths based on $\mathcal {C}_{\text {img}}$ may leak into arteries outside the CoW, whereas the paths based on cost functions using the outputs of the local artery orientation estimator ($\mathcal {C}_{\text {entr}}$, $\mathcal {C}_{\text {orient}}$, $\sum \mathcal {C}_i$) are more restricted to the CoW vasculature. In contrast, paths found using a cost function based on local artery orientations ($\mathcal {C}_{\text {orient}}$) correctly corresponded to the artery of interest, even when arteries were hypoplastic (AcoA, P1(R), P1(L) top row) or aplastic (P1(L) bottom row). However, for some cases, Dijkstra’s algorithm traversed a different nearby artery (PcoA(L) bottom row) or failed to find the correct path for the AcoA.

Quantitative performances of the path extraction using the four cost functions in terms of the Fréchet distance are shown in Fig. 3 (bottom), where lower is better. For all normally developed and hypoplastic artery segments, we found median and IQRs of 3.17 (3.57) mm, 2.26 (1.30) mm, 2.18 (1.33) mm and 2.21 (1.36) mm for $\mathcal {C}_{\text {img}}$, $\mathcal {C}_{\text {entr}}$, $\mathcal {C}_{\text {orient}}$ and $\sum \mathcal {C}_i$ respectively (Fig. 3, bottom left).The bottom right of Fig. 3 shows the Fréchet distances for artery segments categorized by artery diameter. This plot quantitatively confirms that the paths extracted using a cost function beased on local artery orientations are consistently more accurate than the paths extracted with the other cost functions, in particular for smaller arteries. For larger arteries, this performance discrepancy is much smaller. We expect this is because larger arteries are easier to distinguish in the scan and thus result in more more pronounced local minima inside the arteries in cost functions not based on artery orientations. In addition, we observed that combining the three cost functions into the cost function $\sum \mathcal {C}_i$ does not result in more accurate centerlines than the cost function based on local artery orientations alone. Hence, we used the paths extracted using $\mathcal {C}_{\text {orient}}$ as input for subsequent graph reconstruction steps.

Figure 4 shows a comparison between the trajectories extracted with the 3D U-Net and our proposed method, where we used $\mathcal {C}_{\text {orient}}$ for our path extraction. Our method yields a clean, labeled graph representation of the CoW, while the segments from the voxel-masks do not form a clean graph and are unlabeled. While 3D U-Net trajectories for hypoplastic arteries are often discontinuous, our method ensures continuous segments by design but sometimes tracks the wrong artery in hypoplastic cases. We visually assessed these two errors for both methods per artery in the full test set (*N*=40), excluding aplastic artery segments. Table 1 shows that the 3D U-Net achieves over 90% continuous trajectory extraction for larger arteries (ACA-A1(L/R) and PCA-P1(L/R)), but only 35% and 55% for smaller arteries (PcoA(L/R)). Our method achieves correct artery trajectories in 70% and 74% of these smaller arteries, and consistently correct trajectories in larger arteries. Thus, our method offers a more reliable approach for continuous and accurate artery trajectory extraction, while also obtaining a clean and labeled graph representation of the CoW vasculature.

**Table 1:** *First row:* Mean and std diameters of artery segments in the test dataset. *Second and third row:* Comparison between CoW artery trajectories extracted using our method with $\mathcal {C}_{\text {orient}}$ and by skeletonizing a voxel-mask obtained with a 3D U-Net on the test set (*N* = 40). We visually assessed for our method if the correct artery was tracked and for the 3D U-Net if the artery segment was continuous. *Last row:* F1 scores of our RF classifier for artery segments extracted using $\mathcal {C}_{\text {orient}}$ in our test dataset (*N*=40).

	ACA-A1 (R)	ACA-A1 (L)	AcoA	PCA-P1 (R)	PCA-P1 (L)	PcoA (R)	PcoA (L)
Diameter (mm) $\mu$, $\sigma$	2.07 ± 0.32	1.99 ± 0.31	1.09 ± 0.46	1.89 ± 0.46	1.93 ± 0.48	1.27 ± 0.56	1.15 ± 0.57
Ours_correct_ (%)	100	100	77	97	97	74	70
3D U-Net_continuous_ (%)	98	100	74	95	90	55	35
F1-score (RF classifier)	0.99	0.99	0.82	0.97	0.96	0.96	0.96

### Graph reconstruction

**Figure 5:**
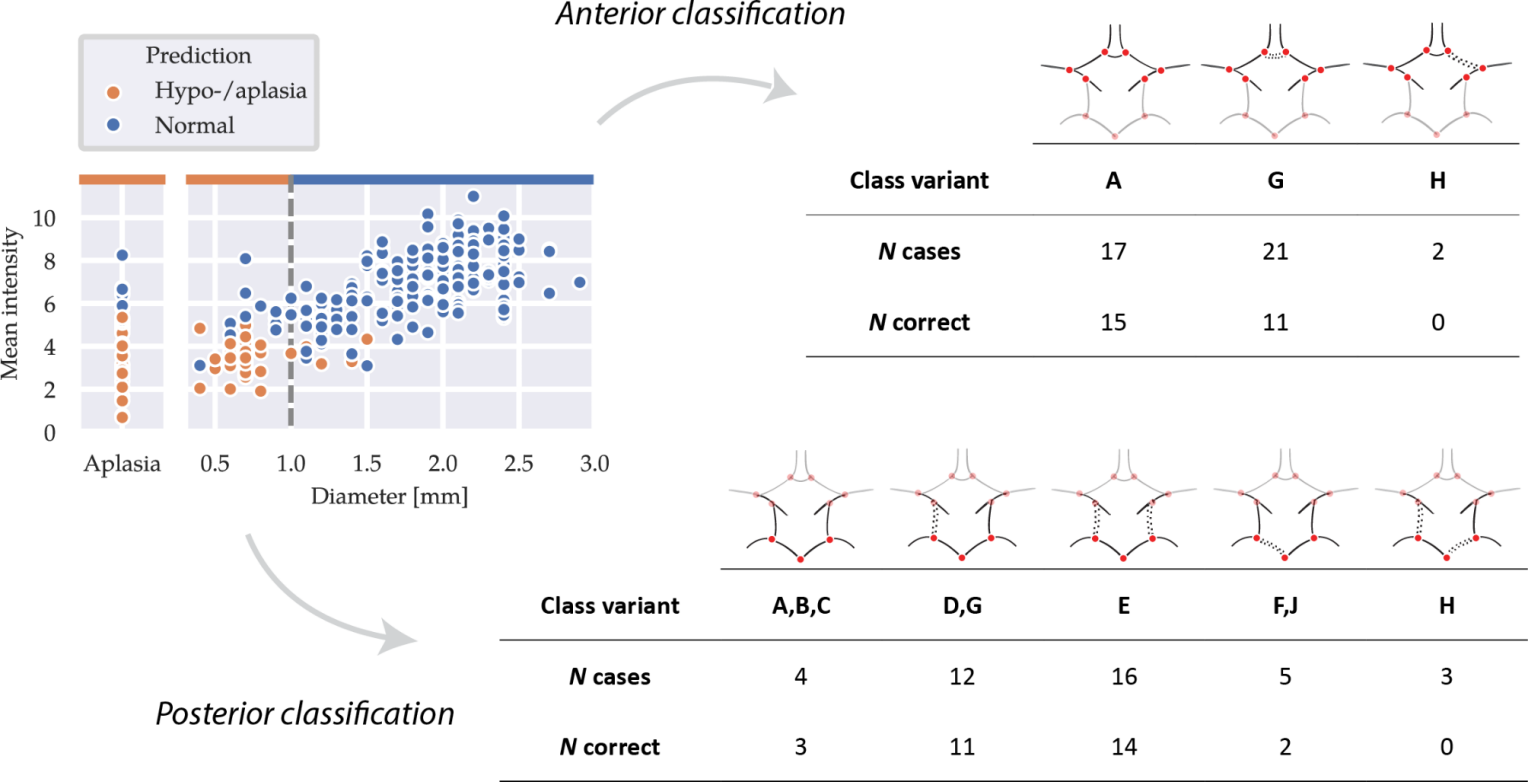
Performance of our graph reconstruction step on the test set. *Left:* predictions of the RF classifier on the arteries in the test set, with measured diameters and the mean intensity along the automatically retrieved path. The dashed line indicates the decision threshold for hypo-/aplastic class. *Upper right:* accuracy of the anterior Lippert classes of our reconstructed graphs. *Lower right:* accuracy of the posterior Lippert classes of our reconstructed graphs.

The RF classifier based on the intensity profiles along the automatically extracted artery trajectories yielded an overall accuracy of 0.91 on all segments in the held-out test set, with F1 scores ranging between 0.82 and 0.99 for individual artery segments (Last row table 1). Among the 25 out of 280 incorrectly classified sections, 12 were AcoAs, 6 were PcoAs, 5 were P1s, and 2 were the A1 segment. Figure 5 (left) shows a clear relation between predicted classes, reference artery diameters, and mean intensities along the tracked paths. We then used these classifications to label the anterior and posterior section of each CoW according to the Lippert classification system, as described in the *Evaluation Criteria* Section. Figure 5 (right) shows the distribution of each Lippert class in our test set and the number of correctly classified cases for each class. The best results were obtained for complete anterior CoW variants (A) and one or two hypo-/aplastic PcoAs in the posterior CoW (D,E,G).

## Discussion

We have proposed a method to automatically extract CoW topology from 3D TOF-MRA scans. We have used a complete CoW as anatomical prior and obtained an (over)complete CoW by connecting automatically detected bifurcation points using Dijkstra’s shortest path algorithm. The proposed orientation-guided cost function resulted in correct and continuous path extraction (i.e., the path follows the artery of interest if present) for 70% and 74% of the left and right PcoAs, respectively. In comparison, the 3D UNet yielded continuous artery centerlines only in 35% and 55% of these PcoAs. Classification of the artery segments yielded F1-scores between 0.82 and 0.99 for all arteries.

Existing graph-based methods to extract CoW topologies often involve segmentation masks used to obtain artery centerlines through skeletonization. However, such voxel-based segmentations can lead to discontinuous centerlines, especially for arteries with diameters of 1 mm or smaller, as shown in Fig. 4 and highlighted by a recently organised CoW segmentation challenge^12^. Their results showed that despite achieving high segmentation accuracy, accurate extraction of the topology remains difficult, with lowest topology match ratios for CoW variants with two (normal or hypoplastic) PcoAs. Figure 5 shows that our method classifies these variants with high accuracy. Lower performances were obtained for hypo-/aplastic PCA-P1 and ACA-A1 segments, which mostly included arteries with diameters near the hypoplasia threshold.

Bifurcation points were detected with a median accuracy below 1.9 mm with outliers of up to 7 mm. As the CoW displays a wide range of anatomical variance, the exact localization of a bifurcation point can be challenging, in particular in the case of hypo- or aplastic arteries. This ambiguity may cause errors in the downstream path extraction step. Moreover, the location of the PcoA bifurcation points displayed the largest variance in the data due to common anatomical configurations such as fetal-type PCAs, hypoplasia, or absence of both PcoAs. This resulted in a lower accuracy of the automatic detection of these bifurcation points.

We found that including the estimated artery orientation in the cost function for path extraction improved the quality of our extracted paths. However, we observe that our method sometimes (partially) tracks the wrong artery. Table 1 shows that this may happen for the PcoA or AcoA. Upon visual inspection, we found that these errors were related to hypoplasia or close proximity to other arteries. As our artery orientation estimator lacks global context, adding anatomical labels from e.g., a multi-label segmentation method to the cost function may mitigate this problem for larger arteries^12^. Additionally, Dijkstra’s algorithm could be exchanged for an alternative shortest path algorithm that allows for the regularization of other features, e.g. tortuosity and length^23^.

Errors in the path extraction step may result in wrong classifications of the artery segments by our RF classifier. Figure 5 shows a strong correlation between the artery diameter and the mean intensity along the automatically localized path. While the RF classifier generally made accurate predictions, some inaccuracies occurred near the hypoplasia threshold of 1.0 mm and in cases of aplasia, particularly for outliers in mean intensity. These outliers imply deviations in the path localization, possibly due to misplaced bifurcation points (e.g. the AcoA in patient 2, Fig. 5) or wrongly traversed artery segments by Dijkstra’s algorithm.

The lack of consensus in the literature regarding the definition of hypoplasia (< 0.8 mm or < 1.0 mm) and classification systems complicates study comparisons and clinical implications. We believe that the method we propose here could improve the reproducibility and interpretability of future studies. Here, we classified reconstructed paths according to the system from^6^ as a proof of concept, where no distinction is made between hypo-/ or aplasia. Rather than strictly adhering to a classification system, one may also consider other edge features in the graph representation that can be used in a downstream learning or analysis task.

Our study has several limitations. First, by using Dijkstra’s algorithm we force the reconstruction of a path, even when the artery of interest is aplastic. We could not validate such path reconstructions since there is no reference for such artery trajectories. Second, we could not validate detected bifurcation points in the absence of a bifurcation. Future work should aim to integrate uncertainty quantification for detecting bifurcation points in aplastic arteries. Third, we were unable to evaluate the intra- and inter-observer variability for each annotation due to the absence of repeated annotations. Fourth, we excluded rare CoW variants such as three or one (azygos) ACA-A2 segments, and other artery duplications. However, our cohort does cover most common anatomical variants. Fifth, our dataset contained scans with different field strengths (1.5T and 3T), future work should assess the effect of field strength on model performances.

We propose a flexible framework for robust personalized extraction of the CoW topology. Our results demonstrate the effectiveness of using scale-invariant features to detect arteries with diameters around 1 mm or smaller. This framework can be customized to any preferred downstream analysis approach (including graph representations) and could facilitate large-scale studies regarding CoW anatomical variation.

## Supplementary Information

Supplementary Information.

## Data Availability

Anonymized data will be made available by request from any qualified investigator. Please contacd.alblas@utwente.nl for any data requests.
