# Supplementary material for: Deep-learning-based extraction of circle of Willis topology with anatomical priors
Source: Sci Rep. 2024 Dec 30;14:31630. doi: 10.1038/s41598-024-80574-0 (PMC11686194; doi:10.1038/s41598-024-80574-0)
Supplement: Supplementary file 1 — Supplementary Information. [file 41598_2024_80574_MOESM1_ESM.pdf]

Appendix A. Data splits

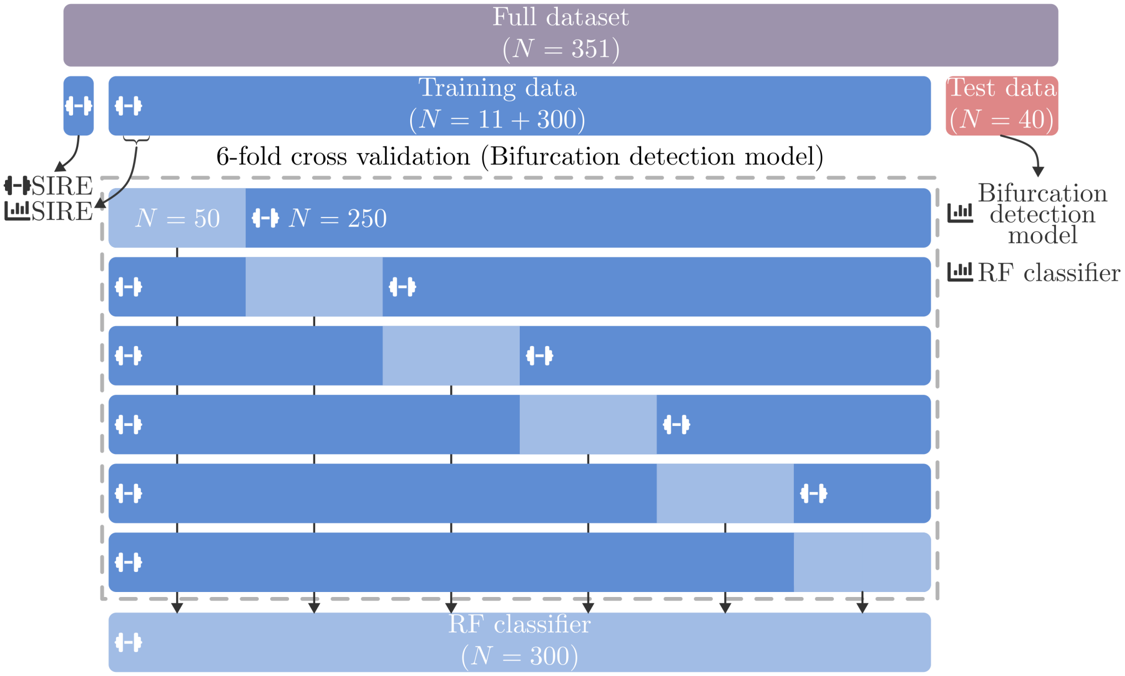

Figure S 1. Flowchart visualizing the data splits for training and testing of SIRE, the bifurcation detection model and the random forest classifier.

Appendix B. Artery bifurcation localization

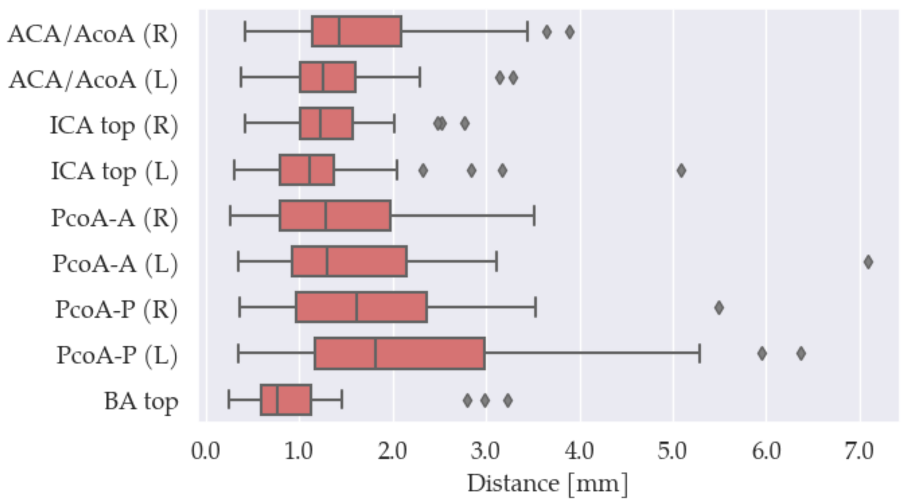

Figure S 2. Performance of artery bifurcation localization per bifurcation point. Euclidean distances between the ground-truth and automatically detected bifurcation points on the test set are reported.
